# Comparison of twelve single-drug regimens for the treatment of type 2 diabetes mellitus

**DOI:** 10.18632/oncotarget.20282

**Published:** 2017-08-16

**Authors:** Shao-Lian Wang, Wen-Bin Dong, Xiao-Lin Dong, Wen-Min Zhu, Fang-Fang Wang, Fang Han, Xin Yan

**Affiliations:** ^1^ Department of Endocrinology, Jinan Central Hospital, Jinan 250013, P.R. China; ^2^ Pharmaceutical Preparation Section, Jinan Central Hospital, Jinan 250013, P.R. China

**Keywords:** type 2 diabetes mellitus, single-drug regimen, effects, randomized controlled trials, network meta-analysis

## Abstract

We performed a network meta-analysis to compare the efficacy of 12 single-drug regimens (Glibenclamide, Glimepiride, Pioglitazone, Rosiglitazone, Repaglinide, Metformin, Sitaglitin, Exenatide, Liraglutide, Acarbose, Benfluorex, and Glipizide) in the treatment of type 2 diabetes mellitus (T2DM). Fifteen relevant randomized controlled trials (RCTs) were included; direct and indirect evidence from these studies was combined, and weighted mean difference (WMD) and surface under the cumulative ranking curves (SUCRAs) were examined to evaluate the monotherapies. Liraglutide was more effective than Glimepiride, Pioglitazone, Sitaglitin, Exenatide, and Glipizide at reducing glycated hemoglobin (HbA1c) levels. In contrast, Acarbose was less effective than Glibenclamide, Glimepiride, Pioglitazone, Rosiglitazone, Repaglinide, Metformin, and Liraglutide at decreasing HbA1c levels. Reductions in fasting plasma glucose (FPG) levels were similar after all treatments. Rosiglitazone was less effective than Glibenclamide and Repaglinide at reducing total cholesterol (TC) levels. High density lipoprotein (HDL), low density lipoprotein (LDL), and triglyceride levels did not differ after treatment with any of the monotherapies. HbA1c and FPG SUCRA values were highest for Liraglutide, while HbA1c and FPG values were lowest for Acarbose, and TC and LDL values were lowest for Rosiglitazone. These results suggest that Liraglutide may be most effective, and Acarbose least effective, at reducing blood glucose levels, while Glibenclamide, Repaglinide, and Metformin may be most effective, and Rosiglitazone least effective, at reducing lipoidemia, in T2DM patients.

## INTRODUCTION

Type 2 diabetes mellitus (T2DM) is a chronic metabolic disease associated with hyperglycemia, which can lead to serious vascular complications [1]. Both genetic and environmental factors contribute to T2DM; up to 25% of first-degree relatives of T2DM patients may suffer from this disease [2], and T2DM is a fast-growing epidemic worldwide [3]. T2DM, which is associated with high rates of hypertension, early microalbuminuria, and dyslipidemia, is increasingly common in obese people and progresses more rapidly in youth than in adults [4]. Chronic complications of T2DM include accelerated development of cardiovascular diseases, end-stage renal disease, loss of visual acuity, and limb amputations, and are the main contributors to morbidity and mortality in T2DM patient [5]. The short-term aim of therapy for hyperglycaemia is to improve blood glucose control while minimizing tolerance to treatment and safety issues; reducing vascular damage is an important long-term objective [6]. Both oral and injectable treatments are currently available for the treatment of T2DM [7].

Various single-drug regimens are commonly used to treat T2DM. Metformin, a biguanide derivate, has pleiotropic effects beyond glucose reduction, including improvement of lipid profiles and reduction microvascular and macrovascular complications associated with T2DM [8]. Pioglitazone, a new thiazolidinedione, is widely to treat T2DM [9] and has been associated with redistribution of body fat, which can predict insulin resistance and adverse drug-related events in patients [10]. Rosiglitazone is an oral hypoglycaemic agent of the thiazolidinedione group that improves plasminogen activity and high-density lipoprotein cholesterol levels in T2DM [11]. Acarbose can be used either alone or in addition to changes in lifestyle to delay development of T2DM in patients with impaired glucose tolerance [12]. Although these drugs have all been crucial for the treatment of T2DM, mortality rates related to poor blood sugar control and accompanying complications remain high in diabetic patients [13]. Additionally, while new targets and treatment methods will likely prove essential in improving outcomes, individual T2DM patients respond differently to different therapies [14]. In this network meta-analysis, we evaluated current clinical data to compare the efficacy of different single-drug regimens in the treatment of T2DM.

## RESULTS

### Baseline characteristics of included studies

In this study, we initially retrieved 1,240 relevant studies, of which 8 were duplicated publications, 201 were letters or reviews, 198 were non-human research, and 268 were not in English and were eliminated. Of the remaining 565 studies, we excluded 178 non-RCT studies, 112 studies unrelated to T2DM, 259 studies that did not use single-drug regimens, and 1 study without sufficient data. Ultimately, 15 eligible RCTs published between 1999 and 2017 that included a total of 3,597 T2DM patients were included in this network meta-analysis [15–29] (Supplementary Figure 1). More patients were treated with Glibenclamide or Repaglinide monotherapies than with the other therapies. Four of the RCTs trials were conducted in Asian patient populations and 11 in Caucasian populations, and 14 trials were two-arm trials while 1 was a three-arm trial. Characteristics of the included studies are shown in Supplementary Appendix Table 1A and Appendix Table 1B; Cochrane bias evaluation is shown in Supplementary Figure 2.

**Table 1:** Weighted mean difference and 95%CI of pairwise meta-analysis in terms of FPG, HbA1c and TC

Studies	Comparison	Pairwise meta-analysis
		WMD (95%CI)
**FPG (mmol/L)**		
1 study	B vs G	−0.50 (−1.68 ∼ 0.68)
1 study	B vs H	−0.33 (−1.18∼ 0.52)
1 study	G vs H	0.17 (−0.95 ∼ 1.29)
2 studies	C vs D	−0.39 (−1.68 ∼ 0.89)
1 study	B vs I	**1.02 (0.42 ∼ 1.62)**
1 study	C vs F	1.30 (−1.21 ∼ 3.81)
1 study	C vs E	0.85 (−0.31 ∼ 2.01)
1 study	C vs J	**-1.03 (−1.94 ∼ −0.12)**
2 studies	A vs E	**-0.37 (−0.67 ∼ −0.07)**
1 study	A vs B	−0.60 (−1.27 ∼ 0.07)
1 study	F vs K	**-0.75 (−1.14 ∼ −0.36)**
1 study	E vs L	**-0.80 (−0.91 ∼ −0.69)**
**HbA1c(%)**		
2 studies	B vs G	−0.15 (−0.30 ∼ 0.01)
1 study	B vs H	−0.30 (−0.74∼ 0.14)
1 study	G vs H	0.19 (−0.09 ∼ 0.47)
2 studies	C vs D	−0.10 (−0.14 ∼ 0.34)
1 study	B vs I	**0.63 (0.36 ∼ 0.90)**
1 study	C vs F	0.50 (−0.92 ∼ 1.92)
1 study	D vs F	0.11 (−0.26 ∼ 0.48)
1 study	C vs E	**0.50 (0.04 ∼ 0.96)**
1 study	C vs J	**-0.81 (−1.33 ∼ −0.29)**
2 studies	A vs E	0.08 (−0.05 ∼ 0.21)
1 study	A vs B	−0.03 (−0.28 ∼ 0.22)
1 study	F vs K	**-0.42 (−0.68 ∼ −0.16)**
1 study	E vs L	**-0.59 (−0.64 ∼ −0.54)**
**TC(mmol/L)**		
1 study	B vs G	−0.44 (−1.45 ∼ 0.57)
1 study	B vs H	0.34 (−0.74∼ 1.42)
1 study	G vs H	0.78 (−0.23 ∼ 1.79)
2 studies	C vs D	**-0.79 (−1.07 ∼ −0.51)**
1 study	C vs F	0.60 (−0.12 ∼ 1.32)
1 study	C vs E	0.72 (−0.61 ∼ 2.05)
2 studies	A vs E	**0.19 (0.08 ∼ 0.30)**
1 study	A vs B	0.02 (−0.15 ∼ 0.19)
1 study	F vs K	**-0.18 (−0.35 ∼ −0.01)**
1 study	E vs L	**-0.07 (−0.10 ∼ −0.04)**

Notes: 95%CI=95% confidence intervals; WMD = Weighted mean difference; FPG= fasting plasma glucose; HbA1c=glycated hemoglobin; TC=total cholesterol; A=Glibenclamide; B=Glimepiride; C=Pioglitazone; D=Rosiglitazone; E=Repaglinide; F=Metformin; G=Sitaglitin; H=Exenatide; I= Liraglutide; J= Acarbose; K=Benfluorex; L=Glipizide; Bold numbers indicate statistically significant differences.

### Pairwise meta-analysis of the efficacy of twelve single-drug T2DM treatment regimens

We carried out direct paired comparisons of the efficacy of 12 single-drug T2DM treatment regimens. Patients who received Glimepiride had higher FPG and HbA1c levels than those who received Liraglutide (FPG: WMD = 1.02, 95%CI = 0.42 ∼ 1.62; HbA1c: WMD = 0.63, 95%CI = 0.36 ∼ 0.90, respectively), which indicated that Liraglutide was more effective than Glimepiride at lowering blood glucose levels in T2DM patients. Compared to those receiving Acarbose, FPG and HbAlc levels were lower in patients who received Pioglitazone (WMD = −1.03, 95%CI = −1.94 ∼ −0.12; WMD = −0.81, 95%CI = −1.33 ∼ −0.29, respectively), which demonstrated that the hypoglycemic effects of Acarbose were relatively poor. Patients taking Glibenclamide had lower LDL levels (WMD = −0.50, 95%CI = −0.66 ∼ −0.34), and those who took Pioglitazone had lower TC and LDL levels (WMD = −0.79, 95%CI = −1.07 ∼ −0.51; WMD = −0.31, 95%CI = −0.54 ∼ −0.08, respectively), than patients treated with Rosiglitazone, indicating that Rosiglitazone was less effective at decreasing blood lipid levels (Table 1 and Table 2). Furthermore, compared to Glipizide, FPG, HbA1c, and TC levels were lower in T2DM patients treated with Repaglinide, which indicated that Repaglinide therapy was more effective. However, FPG and HDL levels were lower in patients treated with Glibenclamide than in those treated with Repaglinide. Additionally, FPG, HbA1c, and TC levels were lower after Metformin treatment than after Benfluorex treatment. HbA1c and HDL levels in T2MD patients treated with Pioglitazone were higher than in those treated with Repaglinide. Compared to Exenatide, LDL and triglyceride levels were higher in patients treated with Sitaglitin (Table 1 and Table 2).

**Table 2:** Weighted mean difference and 95%CI of pairwise meta-analysis in terms of HDL, LDL and triglycerides

Studies	Comparison	Pairwise meta-analysis
		WMD (95%CI)
**HDL (mmol/L)**		
1 study	B vs G	−0.00 (−1.98 ∼ 1.98)
1 study	B vs H	−0.56 (−3.33∼ 2.21)
1 study	G vs H	−0.56 (−2.54 ∼ 1.42)
2 studies	C vs D	0.00 (−0.14 ∼ 0.14)
1 study	C vs F	−0.20 (−0.47 ∼ 0.07)
1 study	D vs F	**0.23 (0.06 ∼ 0.40)**
1 study	C vs E	**0.38 (0.08 ∼ 0.68)**
2 studies	A vs E	**-0.07 (−0.11 ∼ −0.02)**
1 study	A vs B	−0.09 (−0.18 ∼ 0.00)
1 study	F vs K	0.02 (−0.03∼ 0.07)
**LDL (mmol/L)**		
1 study	B vs G	−0.17 (−1.18 ∼ 0.84)
1 study	B vs H	0.78 (−0.23∼ 1.79)
1 study	G vs H	**0.95 (0.02 ∼ 1.88)**
2 studies	C vs D	**-0.31 (−0.54 ∼ −0.08)**
1 study	D vs F	0.32 (−0.32 ∼ 0.96)
1 study	A vs D	**-0.50 (−0.66 ∼ −0.34)**
1 study	C vs E	0.89 (−0.28 ∼ 2.06)
1 study	A vs E	−0.08 (−0.35 ∼ 0.19)
1 study	A vs B	−0.12 (−0.30 ∼ 0.06)
**Triglycerides(mmol/L)**		
1 study	B vs G	−1.39 (−3.45 ∼ 0.67)
1 study	B vs H	0.61 (−1.71 ∼ 2.93)
1 study	G vs H	**2.00 (0.12 ∼ 3.88)**
2 studies	C vs D	0.02 (−0.26 ∼ 0.30)
1 study	C vs F	0.00 (−1.28 ∼ 1.28)
1 study	D vs F	−0.80 (−2.13 ∼ 0.53)
1 study	C vs E	-**5.34 (−9.88 ∼ −0.80)**
2 studies	A vs E	0.25 (−0.07 ∼ 0.57)
1 study	A vs B	−0.26 (−2.21 ∼ 1.69)
1 study	F vs K	−0.02 (−0.23∼ 0.19)
1 study	E vs L	0.00 (−0.03 ∼ 0.03)

Notes: 95%CI=95% confidence intervals; WMD=Weighted mean difference; HDL=High density lipoprotein; LDL=low density lipoprotein; A=Glibenclamide; B=Glimepiride; C=Pioglitazone; D=Rosiglitazone; E=Repaglinide; F=Metformin; G=Sitaglitin; H=Exenatide; I= Liraglutide; J= Acarbose; K=Benfluorex; L=Glipizide; Bold numbers indicate statistically significant differences.

### Evidence network for twelve single-drug T2DM treatment regimens

**Figure 1:**
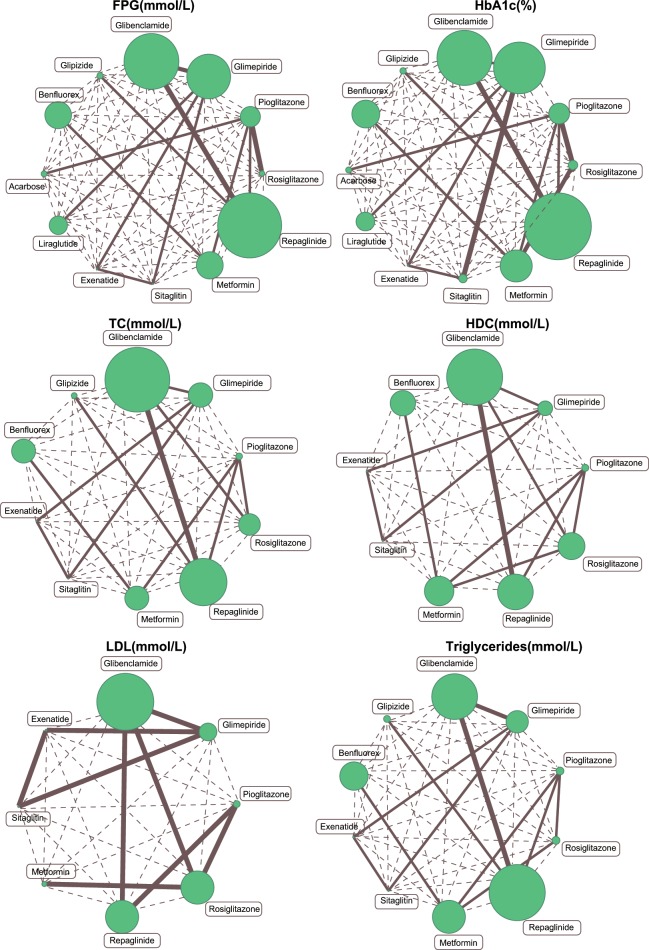
Evidence network plots of FPG, HbA1c, TC, HDL, LDL and triglyceride levels (Note: FPG=fasting plasma glucose; HbA1c = glycated hemoglobin; TC= total cholesterol; HDL= high density lipoprotein; LDL= low density lipoprotein)

The network evidence diagram is shown in Figure 1. It revealed that the majority of T2DM patients received treatment with Glibenclamide or Repaglinide, while fewer patients received Metformin, Acarbose, or Glipizide.

### Inconsistency test for HbA1c, HDL, LDL, and triglycerides among all included studies

**Figure 2:**
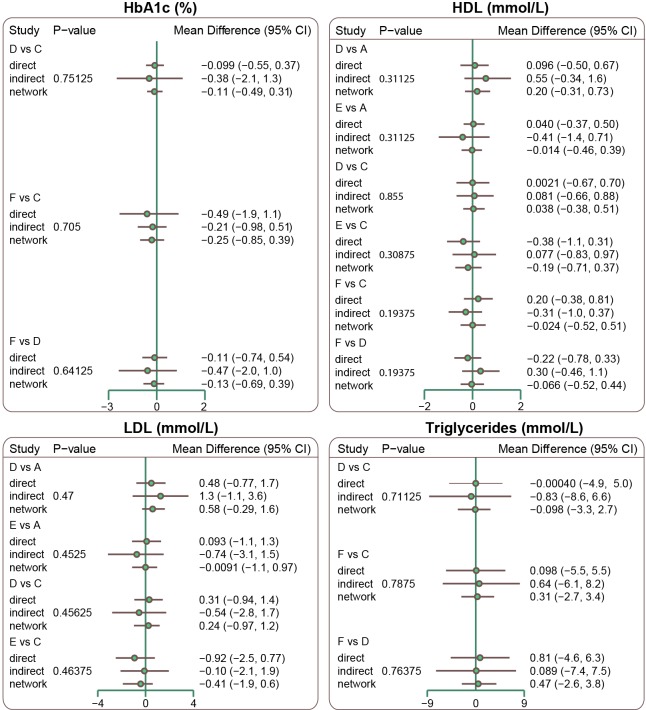
Node splitting graphs of HbA1c, HDL, LDL and triglyceride levels (Note: HbA1c = glycated hemoglobin; HDL = high density lipoprotein; LDL = low density lipoprotein; A = Glibenclamide; B = Glimepiride; C = Pioglitazone; D = Rosiglitazone; E = Repaglinide Benfluorex; L = Glipizide)

We used the node-splitting method to perform an inconsistency test for HbA1c, HDL, LDL, and triglyceride levels, and found that the direct evidence was consistent with the indirect evidence; a consistent model was thus adopted (all *P* > 0.05). (Figure 2)

### Network meta-analysis of twelve single-drug T2DM treatment regimens

**Figure 3:**
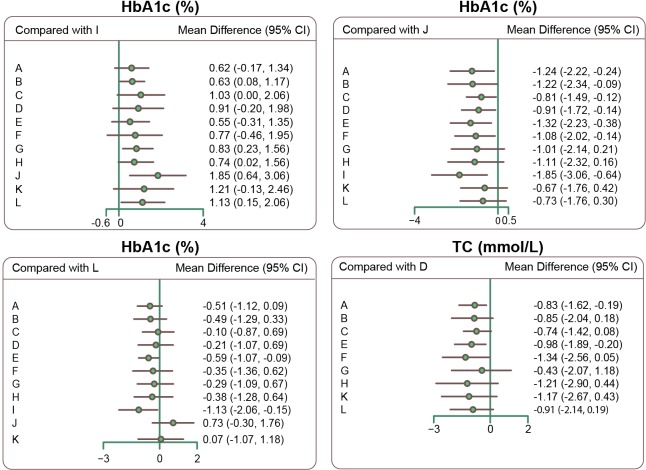
Forest plots of relative relationships for HbA1c and TC (Note: HbA1c = glycated hemoglobin; TC= total cholesterol; A = Glibenclamide; B = Glimepiride; C=Pioglitazone; D=Rosiglitazone; E = Repaglinide; F = Metformin; G = Sitaglitin; H = Exenatide; I = Liraglutide; J = Acarbose; K = Benfluorex; L = Glipizide)

Compared to Liraglutide, HbA1c levels were higher in patients treated with Glimepiride, Pioglitazone, Sitaglitin, Exenatide, or Glipizide, indicating that Liraglutide was more effective at reducing blood glucose levels (WMD = 0.63, 95%CI = 0.08 ∼ 1.17; WMD = 1.03, 95%CI = 0.01 ∼ 2.06; WMD = 0.83, 95%CI = 0.23 ∼ 1.56; WMD = 0.74, 95%CI = 0.02 ∼ 1.56; WMD = 1.13, 95%CI = 0.15 ∼ 2.06, respectively). Compared to Acarbose, HbA1c levels were lower in patients treated with Glibenclamide, Glimepiride, Pioglitazone, Rosiglitazone, Repaglinide, Metformin, or Liraglutide (WMD = −1.24, 95%CI = −2.22 ∼ −0.24; WMD = −1.22, 95%CI = −2.34 ∼ −0.09; WMD = −0.81, 95%CI = −1.49 ∼ −0.12; WMD = −0.91, 95%CI = −1.72 ∼ −0.14; WMD = −1.32, 95%CI = −2.23 ∼ −0.38; WMD = −1.08, 95%CI = −2.02 ∼ −0.14; WMD = −1.85, 95%CI = −3.06 ∼ −0.64, respectively), which suggested that Acarbose was relatively ineffective at reducing blood glucose levels. Compared to Repaglinide, patients who received Glipizide had higher HbA1c levels (WMD = 0.59, 95%CI = 0.09 ∼ 1.07), indicating that Glipizide was less effective at lowing blood glucose levels. However, all drugs were similarly effective in reducing FPG levels. TC levels were lower in patients treated with Glibenclamide or Repaglinide than in those treated with Rosiglitazone (WMD = −0.83, 95%CI = −1.62 ∼ −0.19; WMD = −0.98, 95%CI = −1.89 ∼ −0.20, respectively), which indicated that Rosiglitazone was less effective at decreasing blood lipid levels. Finally, all drugs had similar effects on HDL, LDL, and triglyceride levels in T2DM patients (Table 3; Supplementary Appendix Table 2 and Figure 3).

**Table 3:** Weighted mean difference (WMD) and 95%CI of twelve treatment modalities of HbA1c and TC

**HbA1c(%)**											
**A**	0.01 (−0.49, 0.55)	0.41 (−0.30, 1.16)	0.30 (−0.50, 1.14)	−0.08 (−0.42, 0.29)	0.17 (−0.79, 1.11)	0.23 (−0.37, 0.94)	0.13 (−0.56, 0.94)	−0.62 (−1.34, 0.17)	**1.24 (0.24, 2.22)**	0.58 (−0.49, 1.65)	0.51 (−0.09, 1.12)
-0.01 (−0.55, 0.49)	**B**	0.40 (−0.49, 1.28)	0.28 (−0.69, 1.25)	−0.09 (−0.73, 0.55)	0.14 (−0.95, 1.17)	0.20 (−0.13, 0.65)	0.10 (−0.37, 0.69)	**-0.63 (−1.17, −0.08)**	**1.22 (0.09, 2.34)**	0.56 (−0.63, 1.71)	0.49 (−0.33, 1.29)
-0.41 (−1.16, 0.30)	−0.40 (−1.28, 0.49)	**C**	−0.10 (−0.51, 0.26)	−0.49 (−1.11, 0.13)	−0.25 (−0.91, 0.36)	−0.19 (−1.13, 0.82)	−0.30 (−1.31, 0.79)	**-1.03 (−2.06, −0.00)**	**0.81 (0.12, 1.49)**	0.15 (−0.69, 0.97)	0.10 (−0.69, 0.87)
-0.30 (−1.14, 0.50)	−0.28 (−1.25, 0.69)	0.10 (−0.26, 0.51)	**D**	−0.38 (−1.10, 0.36)	−0.14 (−0.69, 0.38)	−0.08 (−1.08, 1.02)	−0.18 (−1.24, 0.96)	−0.91 (−1.98, 0.20)	**0.91 (0.14, 1.72)**	0.26 (−0.50, 1.01)	0.21 (−0.69, 1.07)
0.08 (−0.29, 0.42)	0.09 (−0.55, 0.73)	0.49 (−0.13, 1.11)	0.38 (−0.36, 1.10)	**E**	0.24 (−0.67, 1.08)	0.30 (−0.40, 1.08)	0.20 (−0.58, 1.08)	−0.55 (−1.35, 0.31)	**1.32 (0.38, 2.23)**	0.66 (−0.38, 1.66)	**0.59 (0.09, 1.07)**
-0.17 (−1.11, 0.79)	−0.14 (−1.17, 0.95)	0.25 (−0.36, 0.91)	0.14 (−0.38, 0.69)	−0.24 (−1.08, 0.67)	**F**	0.07 (−1.02, 1.28)	−0.03 (−1.15, 1.22)	−0.77 (−1.95, 0.46)	**1.08 (0.14, 2.02)**	0.41 (−0.12, 0.96)	0.35 (−0.62, 1.36)
-0.23 (−0.94, 0.37)	−0.20 (−0.65, 0.13)	0.19 (−0.82, 1.13)	0.08 (−1.02, 1.08)	−0.30 (−1.08, 0.40)	−0.07 (−1.28, 1.02)	**G**	−0.10 (−0.61, 0.40)	**-0.83 (−1.56, −0.23)**	1.01 (−0.21, 2.14)	0.36 (−0.96, 1.51)	0.29 (−0.67, 1.09)
-0.13 (−0.94, 0.56)	−0.10 (−0.69, 0.37)	0.30 (−0.79, 1.31)	0.18 (−0.96, 1.24)	−0.20 (−1.08, 0.58)	0.03 (−1.22, 1.15)	0.10 (−0.40, 0.61)	**H**	**-0.74 (−1.56, −0.02)**	1.11 (−0.16, 2.32)	0.46 (−0.91, 1.67)	0.38 (−0.64, 1.28)
0.62 (−0.17, 1.34)	**0.63 (0.08, 1.17)**	**1.03 (0.00, 2.06)**	0.91 (−0.20, 1.98)	0.55 (−0.31, 1.35)	0.77 (−0.46, 1.95)	**0.83 (0.23, 1.56)**	**0.74 (0.02, 1.56)**	**I**	**1.85 (0.64, 3.06)**	1.21 (−0.13, 2.46)	**1.13 (0.15, 2.06)**
**-1.24 (−2.22, −0.24)**	**-1.22 (−2.34, −0.09)**	**-0.81 (−1.49, −0.12)**	**-0.91 (−1.72, −0.14)**	**-1.32 (−2.23, −0.38)**	**-1.08 (−2.02, −0.14)**	−1.01 (−2.14, 0.21)	−1.11 (−2.32, 0.16)	**-1.85 (−3.06, −0.64)**	**J**	−0.67 (−1.76, 0.42)	−0.73 (−1.76, 0.30)
-0.58 (−1.65, 0.49)	−0.56 (−1.71, 0.63)	−0.15 (−0.97, 0.69)	−0.26 (−1.01, 0.50)	−0.66 (−1.66, 0.38)	−0.41 (−0.96, 0.12)	−0.36 (−1.51, 0.96)	−0.46 (−1.67, 0.91)	−1.21 (−2.46, 0.13)	0.67 (−0.42, 1.76)	**K**	−0.07 (−1.18, 1.07)
-0.51 (−1.12, 0.09)	−0.49 (−1.29, 0.33)	−0.10 (−0.87, 0.69)	−0.21 (−1.07, 0.69)	**-0.59 (−1.07, −0.09)**	−0.35 (−1.36, 0.62)	−0.29 (−1.09, 0.67)	−0.38 (−1.28, 0.64)	**-1.13 (−2.06, −0.15)**	0.73 (−0.30, 1.76)	0.07 (−1.07, 1.18)	**L**
**TC(mmol/L)**											
**A**	−0.01 (−0.83, 0.80)	0.11 (−0.65, 1.15)	**0.83 (0.19, 1.62)**	−0.15 (−0.71, 0.42)	−0.49 (−1.76, 1.06)	0.42 (−1.02, 1.94)	−0.35 (−1.88, 1.21)	−0.33 (−1.79, 1.44)	−0.08 (−1.05, 0.92)		
0.01 (−0.80, 0.83)	**B**	0.11 (−0.95, 1.50)	0.85 (−0.18, 2.04)	−0.13 (−1.10, 0.86)	−0.50 (−1.91, 1.29)	0.44 (−0.77, 1.69)	−0.32 (−1.67, 0.97)	−0.32 (−1.96, 1.63)	−0.06 (−1.38, 1.21)		
-0.11 (−1.15, 0.65)	−0.11 (−1.50, 0.95)	**C**	0.74 (−0.08, 1.42)	−0.26 (−1.33, 0.56)	−0.62 (−1.64, 0.46)	0.30 (−1.47, 1.93)	−0.49 (−2.35, 1.25)	−0.44 (−1.78, 0.92)	−0.18 (−1.55, 0.92)		
**-0.83 (−1.62, −0.19)**	−0.85 (−2.04, 0.18)	−0.74 (−1.42, 0.08)	**D**	**-0.98 (−1.89, −0.20)**	−1.34 (−2.56, 0.05)	−0.43 (−2.07, 1.18)	−1.21 (−2.90, 0.44)	−1.17 (−2.67, 0.43)	−0.91 (−2.14, 0.19)		
0.15 (−0.42, 0.71)	0.13 (−0.86, 1.10)	0.26 (−0.56, 1.33)	**0.98 (0.20, 1.89)**	**E**	−0.35 (−1.61, 1.22)	0.56 (−0.95, 2.17)	−0.20 (−1.82, 1.45)	−0.18 (−1.71, 1.62)	0.07 (−0.72, 0.89)		
0.49 (−1.06, 1.76)	0.50 (−1.29, 1.91)	0.62 (−0.46, 1.64)	1.34 (−0.05, 2.56)	0.35 (−1.22, 1.61)	**F**	0.91 (−1.23, 2.75)	0.14 (−2.02, 2.07)	0.18 (−0.66, 0.98)	0.42 (−1.34, 1.89)		
-0.42 (−1.94, 1.02)	−0.44 (−1.69, 0.77)	−0.30 (−1.93, 1.47)	0.43 (−1.18, 2.07)	−0.56 (−2.17, 0.95)	−0.91 (−2.75, 1.23)	**G**	−0.76 (−2.08, 0.46)	−0.74 (−2.75, 1.53)	−0.48 (−2.29, 1.23)		
0.35 (−1.21, 1.88)	0.32 (−0.97, 1.67)	0.49 (−1.25, 2.35)	1.21 (−0.44, 2.90)	0.20 (−1.45, 1.82)	−0.14 (−2.07, 2.02)	0.76 (−0.46, 2.08)	**H**	0.04 (−2.03, 2.36)	0.27 (−1.59, 2.07)		
0.33 (−1.44, 1.79)	0.32 (−1.63, 1.96)	0.44 (−0.92, 1.78)	1.17 (−0.43, 2.67)	0.18 (−1.62, 1.71)	−0.18 (−0.98, 0.66)	0.74 (−1.53, 2.75)	−0.04 (−2.36, 2.03)	**K**	0.25 (−1.69, 1.96)		
0.08 (−0.92, 1.05)	0.06 (−1.21, 1.38)	0.18 (−0.92, 1.55)	0.91 (−0.19, 2.14)	−0.07 (−0.89, 0.72)	−0.42 (−1.89, 1.34)	0.48 (−1.23, 2.29)	−0.27 (−2.07, 1.59)	−0.25 (−1.96, 1.69)	**L**		

**Notes:** 95%CI=95% confidence intervals; HbA1c = glycated hemoglobin; TC= total cholesterol; A=Glibenclamide; B=Glimepiride; C= Pioglitazone; D=Rosiglitazone; E= Repaglinide; F= Metformin; G = Sitaglitin; H= Exenatide; I= Liraglutide; J= Acarbose; K= Benfluorex; L= Glipizide; Bold numbers indicate statistically significant differences.

### Cumulative probability ranking of twelve single-drug T2DM treatment regimens

As shown in Table 4, analysis of SUCRA values to determine efficacy of the twelve drug treatments revealed that Liraglutide had the highest SUCRA values for HbA1c and FPG (HbA1c: 81.17%; FPG: 98.17%), while Acarbose had the lowest values (HbA1c: 24.25%; FPG:10.75%). However, Rosiglitazone had the lowest TC and LDL SUCRA values (TC: 15.5%; LDL: 29.25%). The TC SUCRA value was higher for Metformin than for other the regimens (80%). Repaglinide had the highest HDL SUCRA value (73.11%), while Exenatide had the lowest (37.22%). Sitaglitin had the lowest TG SUCRA value (20.3%).

**Table 4:** SUCRA values of twelve treatment modalities under six endpoint outcomes

Treatments	SUCRA values (%)					
	FPG	HbA1c	TC	HDL	LDL	Triglycerides
**A**	**75.83**	**71.17**	52.20	**70.67**	**68.38**	40.00
**B**	58.00	70.58	55.00	**58.44**	58.75	34.50
**C**	45.75	38.92	45.50	49.89	42.88	**83.70**
**D**	34.00	49.50	15.50	44.78	29.25	**86.20**
**E**	65.33	**79.42**	67.70	**73.11**	**69.38**	42.60
**F**	**73.67**	61.17	**80.00**	53.56	48.25	**77.70**
**G**	45.25	48.67	35.10	54.00	46.50	20.30
**H**	48.25	60.33	**71.30**	37.22	**85.38**	46.80
**I**	**81.17**	**98.17**	NR	NR	NR	NR
**J**	24.25	10.75	NR	NR	NR	NR
**K**	55.50	30.75	**68.40**	56.67	NR	76.90
**L**	44.25	31.17	58.70	NR	NR	42.50

Notes: SUCRA=surface under the cumulative ranking curves; NR=not report; FPG=fasting plasma glucose; HbA1c=glycated hemoglobin; TC=total cholesterol; HDL=high density lipoprotein; LDL=low density lipoprotein; A=Glibenclamide; B=Glimepiride; C=Pioglitazone; D=Rosiglitazone; E=Repaglinide; F=Metformin; G=Sitaglitin; H=Exenatide; I= Liraglutide; J= Acarbose; K=Benfluorex; L=Glipizide; Bold font, the SUCRA is relatively higher when compared with other interventions.

### Cluster analysis of SUCRA values of twelve single-drug T2DM treatment regimens

**Figure 4:**
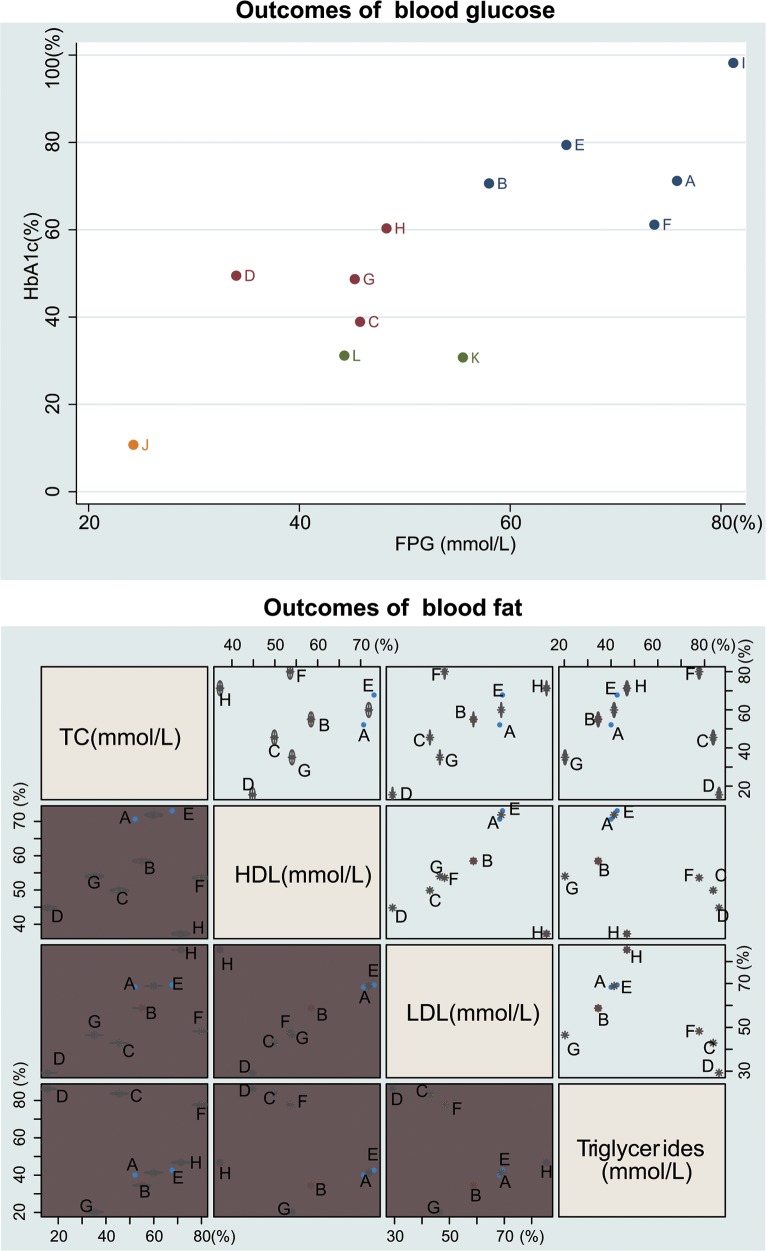
Cluster analysis diagram of twelve single-drug T2DM treatment regimens (Note: FPG = fasting plasma glucose; HbA1c= glycated hemoglobin; TC = total cholesterol; HDL = high density lipoprotein; LDL = low density lipoprotein; A = Glibenclamide; B = Glimepiride; C = Pioglitazone; D = Rosiglitazone; E = Repaglinide; F = Metformin; G = Sitaglitin; H = Exenatide; I = Liraglutide; J = Acarbose; K = Benfluorex; L = Glipizide)

The results of cluster analysis demonstrated that Liraglutide was more effective at reducing blood glucose levels than Glibenclamide, Glimepiride, Repaglinide, and Metformin, all of which were more effective than Acarbose. Glibenclamide and Repaglinide were more effective than the other regimens at reducing HDL and LDL levels, while Metformin was more effective at decreasing TC and triglyceride levels. Rosiglitazone had the lowest efficacy among the twelve regimens in decreasing blood lipid levels (Figure 4).

### Sensitivity analysis

As shown in Supplementary Appendix Table 1A and Appendix Table 1B, there were no significant differences in patient gender and age range among the included studies. Patient body mass index and baseline HbA1c and TC levels were also similar among the included studies, except for Gudipaty *et al*. (2014). We therefore conducted a sensitivity analysis by removing the Gudipaty *et al*. (2014) study and performing the statistical analysis, sorting the interventions, and calculations of cumulative probability rankings again. The rankings of the interventions were largely unchanged in the repeated analysis, indicating that the Gudipaty *et al*. (2004) study did not have a significant impact on our conclusions (Supplementary Appendix Table 3).

### Meta-regression analysis

Because the included studies were conducted in Asian, Caucasian, and mixed patient populations, meta-regression analysis was performed for each outcome. All interventions were then sorted again and cumulative probability rankings were calculated. With the exception of TC levels, there were no significant differences in the intervention rankings for any outcomes before and after meta-regression analysis. Patient ethnicity thus had minimal effects on the results of this study (Supplementary Appendix Table 4).

## DISCUSSION

In this study, we evaluated the efficacy oftwelve single-drug regimens in the treatment of T2DM using pairwise and network meta-analysis. Our results suggest that Liraglutide is more effective than Acarbose at reducing blood glucose levels in T2DM patients. In addition, Glibenclamide, Repaglinide, and Metformin were more effective than Rosiglitazone at decreasing lipoidemia in these patients.

Cumulative probability rankings of the twelve single-drug regimens indicated that Repaglinide had the highest SUCRA value for HDL, while Exenatide had the lowest. However, Sitaglitin had the lowest SUCRA value for TG. A previous study revealed that Repaglinide can be used in both mono- and combined therapies for the treatment of both fasting and postprandial hyperglycemia in T2DM patients [30]. Another study found that Exenatide-treated patients lost a mean ± SD of 3.0 ± 7.33 kg body weight, Sitagliptin-treated patients lost 1.1 ± 5.39 kg body weight, and insulin-treated patients gained 0.6 ± 9.49 kg body weight [31].

Network meta-analysis revealed that HbA1c levels were higher after treatment with Glipizide compared to Repaglinide, indicating that Glipizide was less effective at lowing blood glucose levels. Repaglinide, a prandial glucose regulator, is an effective and safe treatment for T2DM patients, and is more effective than Glipizide at controlling HbA1c and FBG levels overall and in OHA-naive patients [28].

Pairwise meta-analysis revealed that FPG, HbA1c, and TC levels were lower after Metformin treatment than after Benfluorex treatment. Metformin decreases the risk of hepatocellular carcinoma in patients with T2DM by suppressing hepatic glucose output and increasing peripheral glucose uptake and utilization [32, 33]. Another study demonstrated that Metformin and Repaglinide both reduced FPG and HbA1c levels, although Repaglinide induced a larger decrease in HbA1c levels [33]. In addition, HbA1c and HDL levels were higher in T2MD patients treated with Pioglitazone than in those treated with Repaglinide. Pioglitazone improved blood sugar control in T2DM patients by increasing insulin sensitivity and inhibiting lipid peroxidation, while Repaglinide reduced lipid peroxidation and increased total anti-oxidative capacity [34]. In addition, compared to Exenatide, LDL and TC levels were higher after Sitaglitin treatment. A previous study demonstrated that Sitaglitin monotherapy increased insulinogenic index after 52 weeks of treatment [35]. In another evaluation of different T2DM therapies, Exenatide had the most beneficial effect on patient weight, followed by Sitagliptin [31].

While network meta-analyses can provide information to guide clinical decisions, reduce the need for replication of clinical trials, and identify areas that require further study [36], some limitations of this meta-analysis should be considered. The number of included studies was relatively small, and the data and information that could be extracted from these studies were limited. Our findings should therefore be confirmed in additional studies, and the identification of new treatments for T2DM should be a goal of future research.

In conclusion, the present network meta-analysis revealed that Liraglutide may be more effective than other treatments at reducing blood glucose levels in T2DM patients, while Rosiglitazone might be less effective than other drugs at decreasing lipoidemia. Our comprehensive comparison of the efficacy of twelve single-drug regimens may be valuable for guiding clinical decisions made by physicians treating T2DM patients.

## MATERIALS AND METHODS

### Search strategy

The PubMed, EMBASE, and Cochrane library English databases were searched to identify articles published from the inception of each database through February 2017. Manual searches of the reference lists of initially identified studies were also performed. The following keywords combined with free words were used to conduct the search: T2DM, NIDDM, maturity-onset diabetes, diabetes mellitus, noninsulin-dependent, noninsulin-dependent diabetes mellitus, adult-onset diabetes mellitus, ketosis-resistant, maturity-onset diabetes mellitus, MODY, drug therapy, therapy, drug, pharmacotherapy, single-drug, Glibenclamide, Gliclazide, Glipizide, Glimepiride, Metformin, Phenformin, Acarbose, Voglibose, Miglitol, Troglitagone, Gliquidone, Pioglitazone, Rosiglitazone, Repaglinide, Nateglinide, randomized controlled trial, randomized, randomization, double-blind method, placebo, controlled clinical trial, etc.

### Inclusion and exclusion criteria

The inclusion criteria were: (1) studies were randomized controlled trials (RCTs); (2) single-drug regimens (including Glibenclamide, Glimepiride, Pioglitazone, Rosiglitazone, Repaglinide, Metformin, Sitaglitin, Exenatide, Liraglutide, Acarbose, Benfluorex, or Glipizide) were used to treat T2DM; (3) study subjects were 40- to 80-year old patients diagnosed with T2DM; (4) FPG, HbA1c, TC, high density lipoprotein (HDL), LDL, or triglycerides were measured to evaluate outcome. The exclusion criteria were: (1) patients treated with insulin or who had diabetic complications that required treatment; (2) patients with severe deficiencies in liver and kidney function; (3) patients with cardiovascular disease within 3 months of the study; (4) patients who were pregnant or lactating women; (5) patients with severe hypertension; (6) available experimental data was incomplete; (7) non-RCTs; (8) duplicated publications; (9) conference reports, systematic reviews or summaries; (10) studies published in languages other than English.

### Data extraction and quality assessment

Two researchers independently extracted data from the included studies using a standardized data collection form. Disputes regarding data extraction were discussed and negotiated by several researchers until a consensus was reached. Cochrane Collaboration's tool was used to assess the risk of bias in the randomized controlled trials [37]. The tool included six domains: random assignment, allocation concealment, blinding, loss outcome data, choosing outcome reports, and other biases. The assessment involved assigning “yes,” “no,” or “unclear” judgements for each domain to designate low, high, or unclear risks of bias, respectively. Studies with zero or one domain designated “unclear” or “no” were classified as having a low risk of bias, those with two or three domains designated “unclear” or “no” were classified as having a moderate risk of bias, and those with four or more domains designated “unclear” or “no” were classified as having a high risk of bias [38]. Quality assessment and investigation of publication bias were conducted using Review Manager 5 (RevMan 5.2.3, Cochrane Collaboration, Oxford, UK).

### Statistical methods

We performed traditional pairwise meta-analyses for studies that directly compared different treatment arms. Pooled estimates of weighted mean differences (WMD) and 95% confidence intervals (CIs) are reported. Chi-square test and I-square tests were employed to test heterogeneity among the studies [39]. R 3.2.1 software was used to draw a network diagram in which each node represents an intervention measure, the size of node represents sample size, and the thickness of the line between nodes represent the number of studies included. Bayesian network meta-analyses were performed to compare different interventions to each other. Each analysis was based on non-informative priors for effect sizes and precision. After four chains and a 20,000-simulation burn-in phase, convergence and lack of auto correlation were investigated and confirmed; eventually, direct probability statements were derived from an additional 50,000-simulation phase [40]. The study used the node-splitting method to evaluate the consistency of direct and indirect evidence. Node-splitting results for which *P* > 0.05 were analyzed using the consistency model [41]. To assist in interpreting WMDs, we calculated the probability that each intervention was the most effective or safest treatment method by adopting a Bayesian approach using probability values summarized as the surface under the cumulative ranking curve (SUCRA); the larger the SUCRA value, the better the rank for that intervention [42, 43]. Cluster analyses were used to group the treatments according to their similarity with regard to both outcomes [42]. All computations were carried out using the R (V.3.2.1) package gemtc (V.0.6) as well as the Markov Chain Monte Carlo engine Open BUGS (V.3.4.0).

## SUPPLEMENTARY MATERIALS FIGURES AND TABLES







## References

[1] Hanefeld M, Raccah D, Monnier L (2017). Individualized, patient-centered use of lixisenatide for the treatment of type 2 diabetes mellitus. Expert Opin Drug Metab Toxicol.

[2] Qiao YC, Shen J, He L, Hong XZ, Tian F, Pan YH, Liang L, Zhang XX, Zhao HL (2016). Changes of Regulatory T Cells and of Proinflammatory and Immunosuppressive Cytokines in Patients with Type 2 Diabetes Mellitus: A Systematic Review and Meta-Analysis. J Diabetes Res.

[3] Wang Z, Wang J, Chan P (2013). Treating type 2 diabetes mellitus with traditional Chinese and Indian medicinal herbs. Evid Based Complement Alternat Med.

[4] Narasimhan S, Weinstock RS (2014). Youth-onset type 2 diabetes mellitus: lessons learned from the TODAY study. Mayo Clin Proc.

[5] Kao KT, Sabin MA (2016). Type 2 diabetes mellitus in children and adolescents. Aust Fam Physician.

[6] Conget I, Mauricio D, Ortega R, Detournay B, CHADIG study investigators (2016). Characteristics of patients with type 2 diabetes mellitus newly treated with GLP-1 receptor agonists (CHADIG Study): a cross-sectional multicentre study in Spain. BMJ Open.

[7] Marin-Penalver JJ, Martin-Timon I, Sevillano-Collantes C, Del Canizo-Gomez FJ (2016). Update on the treatment of type 2 diabetes mellitus. World J Diabetes.

[8] Napolitano A, Miller S, Nicholls AW, Baker D, Van Horn S, Thomas E, Rajpal D, Spivak A, Brown JR, Nunez DJ (2014). Novel gut-based pharmacology of metformin in patients with type 2 diabetes mellitus. PLoS One.

[9] Zhang LF, Pei Q, Yang GP, Zhao YC, Mu YF, Huang Q, Zhu YL (2014). The effect of IGF2BP2 gene polymorphisms on pioglitazone response in Chinese type 2 diabetes patients. Pharmacology.

[10] Basu A, Basu R, Pattan V, Rizza RA, Jensen MD (2010). Meal fat storage in subcutaneous adipose tissue: comparison of pioglitazone and glipizide treatment of type 2 diabetes. Obesity (Silver Spring).

[11] Mustaffa N, Ibrahim S, Abdullah WZ, Yusof Z (2011). Add-on rosiglitazone therapy improves plasminogen activity and high-density lipoprotein cholesterol in type 2 diabetes mellitus. Blood Coagul Fibrinolysis.

[12] Chiasson JL, Josse RG, Gomis R, Hanefeld M, Karasik A, Laakso M, STOP-NIDDM Trial Research Group (2002). Acarbose for prevention of type 2 diabetes mellitus: the STOP-NIDDM randomised trial. Lancet.

[13] Ricks J, Molnar MZ, Kovesdy CP, Shah A, Nissenson AR, Williams M, Kalantar-Zadeh K (2012). Glycemic control and cardiovascular mortality in hemodialysis patients with diabetes: a 6-year cohort study. Diabetes.

[14] Safavi M, Foroumadi A, Abdollahi M (2013). The importance of synthetic drugs for type 2 diabetes drug discovery. Expert Opin Drug Discov.

[15] Kondo Y, Harada N, Hamasaki A, Kaneko S, Yasuda K, Ogawa E, Harashima S, Yoneda H, Fujita Y, Kitano N, Nakamura Y, Matsuo F, Shinji M (2016). Sitagliptin monotherapy has better effect on insulinogenic index than glimepiride monotherapy in Japanese patients with type 2 diabetes mellitus: a 52-week, multicenter, parallel-group randomized controlled trial. Diabetol Metab Syndr.

[16] Gudipaty L, Rosenfeld NK, Fuller CS, Gallop R, Schutta MH, Rickels MR (2014). Effect of exenatide, sitagliptin, or glimepiride on beta-cell secretory capacity in early type 2 diabetes. Diabetes Care.

[17] ur Rahman I, Idrees M, Salman M, Khan RU, Khan MI, Amin F, Jan NU (2012). A comparison of the effect of glitazones on serum sialic acid in patients with type 2 diabetes. Diab Vasc Dis Res.

[18] Vijay SK, Mishra M, Kumar H, Tripathi K (2009). Effect of pioglitazone and rosiglitazone on mediators of endothelial dysfunction, markers of angiogenesis and inflammatory cytokines in type-2 diabetes. Acta Diabetol.

[19] Garber A, Henry R, Ratner R, Garcia-Hernandez PA, Rodriguez-Pattzi H, Olvera-Alvarez I, Hale PM, Zdravkovic M, Bode B, LEAD-3 (Mono) Study Group (2009). Liraglutide versus glimepiride monotherapy for type 2 diabetes (LEAD-3 Mono): a randomised, 52-week, phase III, double-blind, parallel-treatment trial. Lancet.

[20] Mori K, Emoto M, Araki T, Yokoyama H, Lee E, Teramura M, Koyama H, Shoji T, Inaba M, Nishizawa Y (2008). Effects of pioglitazone on serum fetuin-A levels in patients with type 2 diabetes mellitus. Metabolism.

[21] Stocker DJ, Taylor AJ, Langley RW, Jezior MR, Vigersky RA (2007). A randomized trial of the effects of rosiglitazone and metformin on inflammation and subclinical atherosclerosis in patients with type 2 diabetes. Am Heart J.

[22] Hanefeld M, Patwardhan R, Jones NP, Rosiglitazone Clinical Trials Study Group (2007). A one-year study comparing the efficacy and safety of rosiglitazone and glibenclamide in the treatment of type 2 diabetes. Nutr Metab Cardiovasc Dis.

[23] Jovanovic L, Hassman DR, Gooch B, Jain R, Greco S, Khutoryansky N, Hale PM (2004). Treatment of type 2 diabetes with a combination regimen of repaglinide plus pioglitazone. Diabetes Res Clin Pract.

[24] Masica DN, Kotsanos JG, Beasley CM, Potvin JH (1992). Trend in suicide rates since fluoxetine introduction. Am J Public Health.

[25] Esposito K, Giugliano D, Nappo F, Marfella R, Campanian Postprandial Hyperglycemia Study Group (2004). Regression of carotid atherosclerosis by control of postprandial hyperglycemia in type 2 diabetes mellitus. Circulation.

[26] Martin S, Kolb H, Beuth J, van Leendert R, Schneider B, Scherbaum WA (2003). Change in patients’ body weight after 12 months of treatment with glimepiride or glibenclamide in Type 2 diabetes: a multicentre retrospective cohort study. Diabetologia.

[27] Del Prato S, Erkelens DW, Leutenegger M (2003). Six-month efficacy of benfluorex vs. placebo or metformin in diet-failed type 2 diabetic patients. Acta Diabetol.

[28] Madsbad S, Kilhovd B, Lager I, Mustajoki P, Dejgaard A, Scandinavian Repaglinide Group (2001). Comparison between repaglinide and glipizide in Type 2 diabetes mellitus: a 1-year multicentre study. Diabet Med.

[29] Marbury T, Huang WC, Strange P, Lebovitz H (1999). Repaglinide versus glyburide: a one-year comparison trial. Diabetes Res Clin Pract.

[30] Johansen OE, Birkeland KI (2007). Defining the role of repaglinide in the management of type 2 diabetes mellitus: a review. Am J Cardiovasc Drugs.

[31] Horton ES, Silberman C, Davis KL, Berria R (2010). Weight loss, glycemic control, and changes in cardiovascular biomarkers in patients with type 2 diabetes receiving incretin therapies or insulin in a large cohort database. Diabetes Care.

[32] Harris K, Smith L (2013). Safety and efficacy of metformin in patients with type 2 diabetes mellitus and chronic hepatitis C. Ann Pharmacother.

[33] Ma J, Liu LY, Wu PH, Liao Y, Tao T, Liu W (2014). Comparison of metformin and repaglinide monotherapy in the treatment of new onset type 2 diabetes mellitus in China. J Diabetes Res.

[34] Gumieniczek A, Komsta L, Chehab MR (2011). Effects of two oral antidiabetics, pioglitazone and repaglinide, on aconitase inactivation, inflammation and oxidative/nitrosative stress in tissues under alloxan-induced hyperglycemia. Eur J Pharmacol.

[35] Franzblau SG, White KE, O'sullivan JF (1989). Structure-activity relationships of tetramethylpiperidine-substituted phenazines against Mycobacterium leprae in vitro. Antimicrob Agents Chemother.

[36] Borba HH, Wiens A, Steimbach LM, Perlin CM, Tonin FS, Pedroso ML, Fernandez-Llimos F, Pontarolo R (2017). Network meta-analysis of first- and second-generation protease inhibitors for chronic hepatitis C genotype 1: efficacy based on RVR and SVR 24. Eur J Clin Pharmacol.

[37] Higgins JP, Altman DG, Gotzsche PC, Juni P, Moher D, Oxman AD, Savovic J, Schulz KF, Weeks L, Sterne JA, Cochrane Bias Methods Group, Cochrane Statistical Methods Group (2011). The Cochrane Collaboration's tool for assessing risk of bias in randomised trials. BMJ.

[38] Chung JH, Lee SW (2013). Assessing the quality of randomized controlled urological trials conducted by korean medical institutions. Korean J Urol.

[39] Chen LX, Li YL, Ning GZ, Li Y, Wu QL, Guo JX, Shi HY, Wang XB, Zhou Y, Feng SQ (2015). Comparative efficacy and tolerability of three treatments in old people with osteoporotic vertebral compression fracture: a network meta-analysis and systematic review. PLoS One.

[40] Tu YK, Needleman I, Chambrone L, Lu HK, Faggion CM (2012). A Bayesian network meta-analysis on comparisons of enamel matrix derivatives, guided tissue regeneration and their combination therapies. J Clin Periodontol.

[41] Zhu GQ, Shi KQ, Huang S, Wang LR, Lin YQ, Huang GQ, Chen YP, Braddock M, Zheng MH (2015). Systematic review with network meta-analysis: the comparative effectiveness and safety of interventions in patients with overt hepatic encephalopathy. Aliment Pharmacol Ther.

[42] Chaimani A, Higgins JP, Mavridis D, Spyridonos P, Salanti G (2013). Graphical tools for network meta-analysis in STATA. PLoS One.

[43] Salanti G, Ades AE, Ioannidis JP (2011). Graphical methods and numerical summaries for presenting results from multiple-treatment meta-analysis: an overview and tutorial. J Clin Epidemiol.

