# Supplementary material for: Comparison of twelve single-drug regimens for the treatment of type 2 diabetes mellitus
Source: Oncotarget. 2017 Aug 16;8(42):72700–13. doi: 10.18632/oncotarget.20282 (PMC5641162; doi:10.18632/oncotarget.20282)
Supplement: Supplementary file 1 [file oncotarget-08-72700-s001.pdf]

Comparison of twelve single-drug regimens for the treatment of type 2 diabetes mellitus

SUPPLEMENTARY MATERIALS

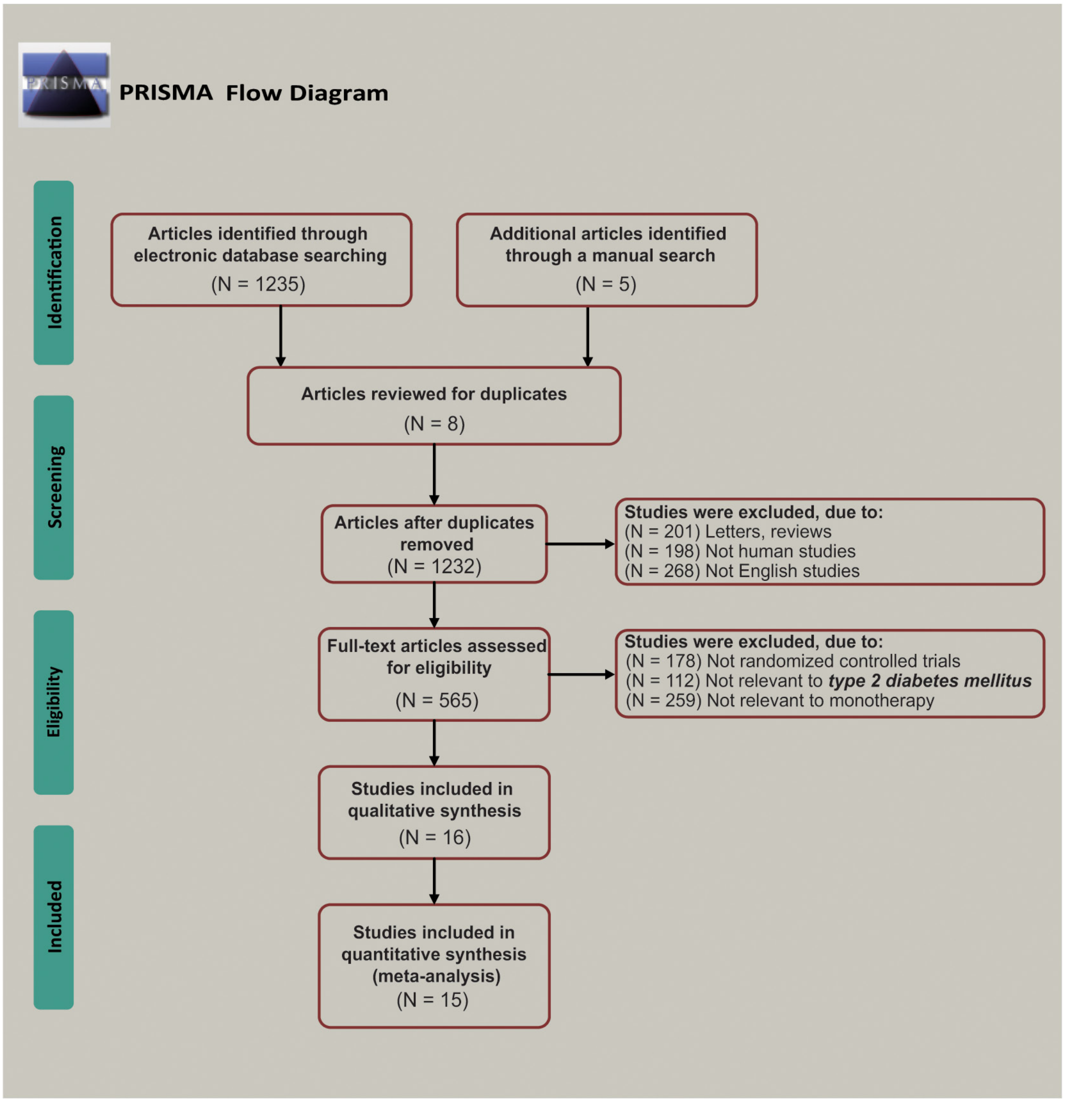

Supplementary Figure 1: Flow chart of the literature search and study selection process.

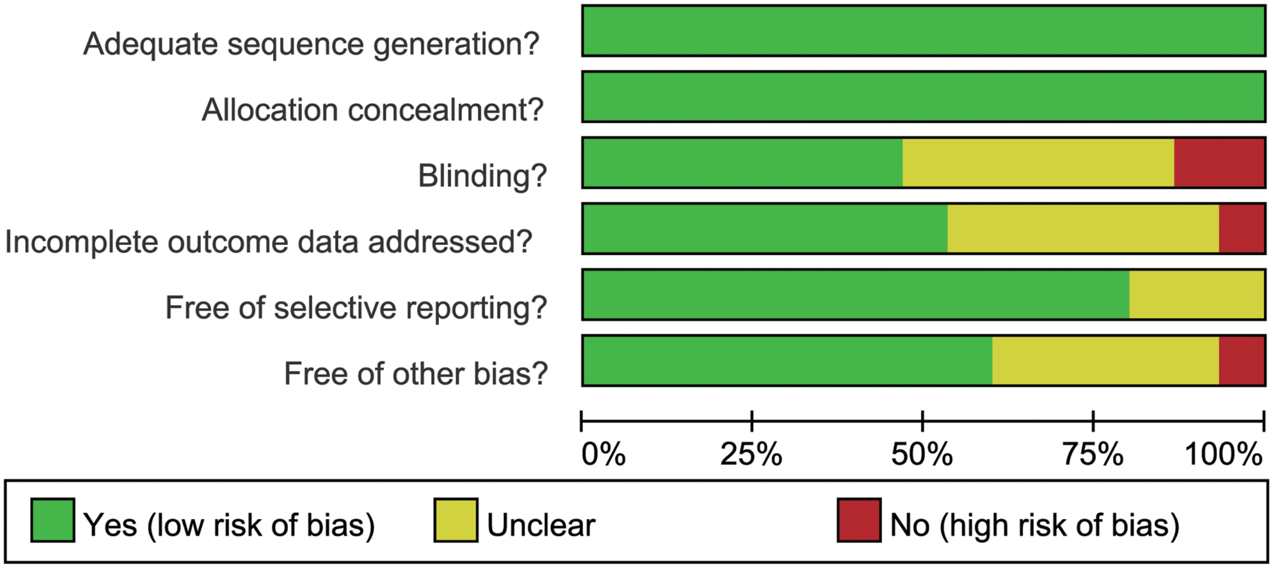

Supplementary Figure 2: Cochrane system bias evaluation of included studies.

Appendix Table 1A: The baseline characteristics for included studies.

Appendix Table 1B: The baseline characteristics for included studies.

See Supplementary File 1

Appendix Table 2: Weighted mean difference (WMD) and 95%CI of twelve treatment modalities of FPG, HDL, LDL and Triglycerides.

See Supplementary File 2

Appendix Table 3: SUCRA values of eleven treatment modalities under six endpoint outcomes after sensitivity analysis

Treatments	SUCRA values (%)					
	FPG	HbA1c	TC	HDL	LDL	Triglycerides
A	**75.10**	**71.00**	53.50	**73.71**	**76.83**	33.88
B	54.60	67.73	55.88	**57.14**	**63.67**	29.62
C	45.30	37.73	44.25	45.57	47.17	**80.88**
D	33.00	48.27	15.62	39.57	30.17	**83.88**
E	65.70	**79.00**	**69.50**	**76.00**	**76.33**	36.25
F	**74.10**	61.09	**80.88**	52.29	56.17	**73.75**
G	NR	60.09	NR	NR	NR	NR
I	**79.60**	**95.73**	NR	NR	NR	NR
J	23.80	11.73	NR	NR	NR	NR
K	55.80	32.00	**68.25**	56.71	NR	73.12
L	43.70	35.00	61.62	NR	NR	36.38

Notes: SUCRA=surface under the cumulative ranking curves; NR=not report; FPG=fasting plasma glucose; HbA1c=glycated hemoglobin; TC=total cholesterol; HDL=high density lipoprotein; LDL=low density lipoprotein; A=Glibenclamide; B=Glimepiride; C=Pioglitazone; D=Rosiglitazone; E=Repaglinide; F=Metformin; G=Sitaglitin; I= Liraglutide; J= Acarbose; K=Benfluorex; L=Glipizide; Bold font, the SUCRA is relatively higher when compared with other interventions.

Appendix Table 4: SUCRA values of twelve treatment modalities under six endpoint outcomes after meta regression analysis

Treatments	SUCRA values (%)					
	FPG	HbA1c	TC	HDL	LDL	Triglycerides
A	**75.46**	**68.74**	54.96	**57.85**	**60.91**	38.13
B	57.81	68.06	58.13	54.50	52.04	35.41
C	45.27	40.47	56.33	53.54	48.76	**82.63**
D	34.19	50.68	48.74	50.75	36.88	**85.30**
E	65.60	**78.36**	19.09	**68.64**	**69.66**	43.44
F	**71.56**	61.47	63.50	54.02	55.10	**77.00**
G	46.23	48.73	**73.51**	**59.43**	46.13	22.14
H	50.16	59.11	40.40	46.13	**80.52**	45.84
I	**80.65**	**95.15**	NR	NR	NR	NR
J	24.37	12.40	NR	NR	NR	NR
K	54.64	32.80	**70.90**	55.14	NR	76.52
L	44.08	34.03	**64.42**	NR	NR	43.59

Notes: SUCRA=surface under the cumulative ranking curves; NR=not report; FPG=fasting plasma glucose; HbA1c=glycated hemoglobin; TC=total cholesterol; HDL=high density lipoprotein; LDL=low density lipoprotein; A=Glibenclamide; B=Glimepiride; C=Pioglitazone; D=Rosiglitazone; E=Repaglinide; F=Metformin; G=Sitaglitin; H=Exenatide; I= Liraglutide; J= Acarbose; K=Benfluorex; L=Glipizide; Bold font, the SUCRA is relatively higher when compared with other interventions.
