# Supplementary material for: Comparison of twelve single-drug regimens for the treatment of type 2 diabetes mellitus
Source: Oncotarget. 2017 Aug 16;8(42):72700–13. doi: 10.18632/oncotarget.20282 (PMC5641162; doi:10.18632/oncotarget.20282)
Supplement: Supplementary file 2 [file oncotarget-08-72700-s002.docx]

**Appendix table 1a. The baseline characteristics for included studies.**

| **First author** | **Year** | **Country** | **Interventions** | | | **Total** | **Sample size** | | | **Gender(M/F)** | | | **Age (years)** | | |
| --- | --- | --- | --- | --- | --- | --- | --- | --- | --- | --- | --- | --- | --- | --- | --- |
|  |  |  | **T1** | **T2** | **T3** |  | **T1** | **T2** | **T3** | **T1** | **T2** | **T3** | **T1** | **T2** | **T3** |
| Kondo Y | 2016 | Japan | Glimepiride | Sitagliptin | - | 133 | 68 | 65 | - | 2.6 | 3.1 | - | 64.0±8.0 | 63.0±9.0 | - |
| Gudipaty L | 2014 | America | Glimepiride | Sitagliptin | Exenatide | 40 | 14 | 12 | 14 | 1.3 | 2 | 1.8 | 52.0±3.0 | 57.0±3.0 | 57.0±2.0 |
| ur Rahman I | 2012 | Pakistan | Pioglitazone | Rosiglitazone | - | 60 | 30 | 30 | - | 0.9 | 0.8 | - | 51.0±5.5 | 50.2± 4.1 | - |
| Vijay SK | 2009 | India | Pioglitazone | Rosiglitazone | - | 40 | 20 | 20 | - | NR | NR | - | 48.1±5.3 | 47.8± 6.0 | - |
| Garber A | 2009 | America | Glimepiride | Liraglutide | - | 495 | 248 | 247 | - | 1.2 | 1.0 | - | 53·4±10.9 | 52.0±10.8 | - |
| Mori K | 2008 | Japan | Pioglitazone | Metformin | - | 19 | 10 | 9 | - | 2.3 | 0.8 | - | 63.0±10.0 | 62.0±6.0 | - |
| Stocker DJ | 2007 | America | Rosiglitazone | Metformin | - | 92 | 45 | 47 | - | 2.5 | 1.1 | - | 64.0±11.0 | 65.0±10.0 | - |
| Hanefeld M | 2007 | Germany | Glibenclamide | Rosiglitazone | - | 392 | 203 | 189 | - | 2.4 | 1.4 | - | 60.1±8.3 | 60.6±9.2 | - |
| Jovanovic L | 2004 | America | Pioglitazone | Repaglinide | - | 123 | 62 | 61 | - | 1.0 | 1.4 | - | 56.2±12.2 | 57.8±13.1 | - |
| Goke B | 2004 | Germany | Pioglitazone | Acarbose | - | 130 | 75 | 55 | - | NR | NR | - | 58.9±9.1 | 58.9±9.1 | - |
| Esposito K | 2004 | Italy | Glibenclamide | Repaglinide | - | 175 | 87 | 88 | - | 1.1 | 1.1 | - | 51.3±5.9 | 52.0±6.4 | - |
| Martin S | 2003 | Germany | Glibenclamide | Glimepiride | - | 520 | 269 | 251 | - | 2.3 | 1.5 | - | 64.9±10.6 | 62.2±11.1 | - |
| Del Prato S | 2003 | Italy | Metformin | Benfluorex | - | 578 | 284 | 294 | - | 1.5 | 1.3 | - | 56.0±9.0 | 56.0±9.0 | - |
| Madsbad S | 2001 | Denmark | Repaglinide | Glipizide | - | 256 | 175 | 81 | - | 1.6 | 1.8 | - | 60.2±8.1 | 62.0±8.8 | - |
| Marbury T | 1999 | America | Glibenclamide | Repaglinide | - | 544 | 182 | 362 | - | 1.9 | 2.0 | - | 58.7±9.0 | 58.3±9.4 | - |

Notes: T = treatment; M = male; F = female.

**Appendix table 1b. The baseline characteristics for included studies.**

| **First author** | **Year** | **Ethnicity** | **Risk of bias** | **Interventions** | | | **BMI (kg/m^2^)** | | | **HbA1c (%)** | | | **TC (mg/dl)** | | |
| --- | --- | --- | --- | --- | --- | --- | --- | --- | --- | --- | --- | --- | --- | --- | --- |
|  |  |  |  | **T1** | **T2** | **T3** | **T1** | **T2** | **T3** | **T1** | **T2** | **T3** | **T1** | **T2** | **T3** |
| Kondo Y | 2016 | Asians | moderate | Glimepiride | Sitagliptin | - | 24.7±3.3 | 24.1±3.8 | - | 7.5±0.5 | 7.4±0.5 | - | NR | NR | - |
| Gudipaty L | 2014 | Caucasians | moderate | Glimepiride | Sitagliptin | Exenatide | 31.0±2.0 | 33.0±1.0 | 33.0±2.0 | 6.7±0.1 | 6.5±0.1 | 6.4±0.1 | 172.0±10.0 | 159.0±12.0 | 168.0±7.0 |
| ur Rahman I | 2012 | Asians | moderate | Pioglitazone | Rosiglitazone | - | 26.0±2.2 | 26.5±2.2 | - | 8.0±0.7 | 8.1±0.9 | - | 207.9±8.1 | 201.0±13.5 | - |
| Vijay SK | 2009 | Asians | moderate | Pioglitazone | Rosiglitazone | - | 32.3±1.4 | 32.0±2.0 | - | 9.3±1.0 | 9.1±0.8 | - | 219.1±38.1 | 212.2±33.6 | - |
| Garber A | 2009 | mixed population | low | Glimepiride | Liraglutide | - | 33.2±5.6 | 32.8±6.3 | - | 8.4±1.2 | 8.3±1.1 | - | NR | NR | - |
| Mori K | 2008 | Asians | moderate | Pioglitazone | Metformin | - | 26.8±3.5 | 28.0±3.8 | - | 8.0±0.8 | 9.5±2.2 | - | 193.4±30.9 | 216.6±19.3 | - |
| Stocker DJ | 2007 | Caucasians | moderate | Rosiglitazone | Metformin | - | 29.4±0.7 | 29.7±0.7 | - | 8.5±0.3 | 8.5±0.2 | - | NR | NR | - |
| Hanefeld M | 2007 | mixed population | moderate | Glibenclamide | Rosiglitazone | - | 28.7±3.9 | 28.7±3.7 | - | 8.2±1.3 | 8.2±1.4 | - | 216.6±12.0 | 220.4±12.8 | - |
| Jovanovic L | 2004 | Caucasians | moderate | Pioglitazone | Repaglinide | - | 32.1±5.3 | 31.2±5.3 | - | 9.1±1.2 | 9.0±1.1 | - | 200.0±50.0 | 204.0±45.0 | - |
| Goke B | 2004 | Caucasians | low | Pioglitazone | Acarbose | - | 30.9±4.9 | 30.9±4.9 | - | 8.9±1.1 | 8.6±1.1 | - | NR | NR | - |
| Esposito K | 2004 | Caucasians | low | Glibenclamide | Repaglinide | - | 28.3±4.1 | 28.5±4.3 | - | 7.4±1.1 | 7.5±1.1 | - | 201.0±36.0 | 197.0±34.0 | - |
| Martin S | 2003 | Caucasians | moderate | Glibenclamide | Glimepiride | - | 30.4±3.3 | 30.6±3.2 | - | 8.1±1.5 | 8.3±1.6 | - | 232.0±42.5 | 232.0±42.5 | - |
| Del Prato S | 2003 | Caucasians | low | Metformin | Benfluorex | - | 29.7±4.2 | 29.9±4.0 | - | 7.4±1.5 | 7.7±1.6 | - | 205.0±34.8 | 205.0±38.7 | - |
| Madsbad S | 2001 | Caucasians | moderate | Repaglinide | Glipizide | - | 28.0±3.5 | 28.0±3.6 | - | 7.3±1.2 | 7.2±1.4 | - | NR | NR | - |
| Marbury T | 1999 | mixed population | moderate | Glibenclamide | Repaglinide | - | 29.1±3.7 | 29.4±3.7 | - | 8.9±1.6 | 8.7±1.7 | - | NR | NR | - |

Notes: T = treatment; M = male; F = female; BMI = body mass index; HbA1c = glycated hemoglobin; TC = total cholesterol; NR = not report.
