# Supplementary material for: Comparison of twelve single-drug regimens for the treatment of type 2 diabetes mellitus
Source: Oncotarget. 2017 Aug 16;8(42):72700–13. doi: 10.18632/oncotarget.20282 (PMC5641162; doi:10.18632/oncotarget.20282)
Supplement: Supplementary file 3 [file oncotarget-08-72700-s003.docx]

| **Appendix table 2. Weighted mean difference (WMD) and 95%CI of twelve treatment modalities of FPG、HDL、LDL and Triglycerides.** | | | | | | | | | | | |
| --- | --- | --- | --- | --- | --- | --- | --- | --- | --- | --- | --- |
| **FPG(mmol/L)** |  |  |  |  |  |  |  |  |  |  |  |
| **A** | 0.60 (-1.20, 2.35) | 1.16 (-1.21, 3.51) | 1.59 (-1.05, 4.22) | 0.32 (-0.90, 1.51) | -0.14 (-3.93, 3.66) | 1.08 (-1.60, 3.76) | 0.91 (-1.69, 3.45) | -0.41 (-2.95, 2.08) | 2.22 (-0.85, 5.24) | 0.57 (-3.61, 4.82) | 1.10 (-1.00, 3.20) |
| -0.60 (-2.35, 1.20) | **B** | 0.57 (-2.46, 3.63) | 0.99 (-2.25, 4.26) | -0.28 (-2.49, 1.91) | -0.74 (-5.04, 3.54) | 0.49 (-1.52, 2.58) | 0.32 (-1.54, 2.18) | -1.01 (-2.83, 0.75) | 1.62 (-1.96, 5.23) | -0.02 (-4.63, 4.60) | 0.52 (-2.31, 3.27) |
| -1.16 (-3.51, 1.21) | -0.57 (-3.63, 2.46) | **C** | 0.45 (-0.83, 1.61) | -0.84 (-2.89, 1.19) | -1.31 (-4.33, 1.71) | -0.09 (-3.72, 3.56) | -0.25 (-3.76, 3.24) | -1.60 (-5.08, 1.98) | 1.05 (-0.92, 2.93) | -0.59 (-3.95, 2.88) | -0.02 (-2.73, 2.57) |
| -1.59 (-4.22, 1.05) | -0.99 (-4.26, 2.25) | -0.45 (-1.61, 0.83) | **D** | -1.26 (-3.64, 1.12) | -1.75 (-4.94, 1.54) | -0.50 (-4.29, 3.33) | -0.69 (-4.37, 3.05) | -2.01 (-5.76, 1.79) | 0.62 (-1.63, 2.89) | -1.05 (-4.59, 2.72) | -0.47 (-3.40, 2.44) |
| -0.32 (-1.51, 0.90) | 0.28 (-1.91, 2.49) | 0.84 (-1.19, 2.89) | 1.26 (-1.12, 3.64) | **E** | -0.47 (-4.03, 3.14) | 0.76 (-2.18, 3.70) | 0.59 (-2.19, 3.47) | -0.75 (-3.55, 2.09) | 1.88 (-0.91, 4.69) | 0.25 (-3.70, 4.26) | 0.79 (-0.90, 2.53) |
| 0.14 (-3.66, 3.93) | 0.74 (-3.54, 5.04) | 1.31 (-1.71, 4.33) | 1.75 (-1.54, 4.94) | 0.47 (-3.14, 4.03) | **F** | 1.23 (-3.52, 5.99) | 1.07 (-3.61, 5.69) | -0.28 (-4.92, 4.41) | 2.35 (-1.20, 5.89) | 0.72 (-1.00, 2.45) | 1.28 (-2.73, 5.22) |
| -1.08 (-3.76, 1.60) | -0.49 (-2.58, 1.52) | 0.09 (-3.56, 3.72) | 0.50 (-3.33, 4.29) | -0.76 (-3.70, 2.18) | -1.23 (-5.99, 3.52) | **G** | -0.16 (-2.19, 1.83) | -1.51 (-4.23, 1.12) | 1.14 (-2.90, 5.16) | -0.50 (-5.47, 4.50) | 0.04 (-3.40, 3.45) |
| -0.91 (-3.45, 1.69) | -0.32 (-2.18, 1.54) | 0.25 (-3.24, 3.76) | 0.69 (-3.05, 4.37) | -0.59 (-3.47, 2.19) | -1.07 (-5.69, 3.61) | 0.16 (-1.83, 2.19) | **H** | -1.33 (-3.93, 1.26) | 1.32 (-2.72, 5.26) | -0.35 (-5.32, 4.68) | 0.22 (-3.14, 3.53) |
| 0.41 (-2.08, 2.95) | 1.01 (-0.75, 2.83) | 1.60 (-1.98, 5.08) | 2.01 (-1.79, 5.76) | 0.75 (-2.09, 3.55) | 0.28 (-4.41, 4.92) | 1.51 (-1.12, 4.23) | 1.33 (-1.26, 3.93) | **I** | 2.64 (-1.35, 6.71) | 1.00 (-3.92, 5.98) | 1.55 (-1.80, 4.85) |
| -2.22 (-5.24, 0.85) | -1.62 (-5.23, 1.96) | -1.05 (-2.93, 0.92) | -0.62 (-2.89, 1.63) | -1.88 (-4.69, 0.91) | -2.35 (-5.89, 1.20) | -1.14 (-5.16, 2.90) | -1.32 (-5.26, 2.72) | -2.64 (-6.71, 1.35) | **J** | -1.67 (-5.57, 2.31) | -1.09 (-4.38, 2.14) |
| -0.57 (-4.82, 3.61) | 0.02 (-4.60, 4.63) | 0.59 (-2.88, 3.95) | 1.05 (-2.72, 4.59) | -0.25 (-4.26, 3.70) | -0.72 (-2.45, 1.00) | 0.50 (-4.50, 5.47) | 0.35 (-4.68, 5.32) | -1.00 (-5.98, 3.92) | 1.67 (-2.31, 5.57) | **K** | 0.55 (-3.87, 4.83) |
| -1.10 (-3.20, 1.00) | -0.52 (-3.27, 2.31) | 0.02 (-2.57, 2.73) | 0.47 (-2.44, 3.40) | -0.79 (-2.53, 0.90) | -1.28 (-5.22, 2.73) | -0.04 (-3.45, 3.40) | -0.22 (-3.53, 3.14) | -1.55 (-4.85, 1.80) | 1.09 (-2.14, 4.38) | -0.55 (-4.83, 3.87) | **L** |
| **HDL(mmol/L)** |  |  |  |  |  |  |  |  |  |  |  |
| **A** | 0.09 (-0.53, 0.67) | 0.16 (-0.36, 0.73) | 0.19 (-0.28, 0.74) | -0.02 (-0.43, 0.35) | 0.14 (-0.49, 0.84) | 0.24 (-2.05, 2.25) | 0.81 (-2.10, 3.68) | 0.12 (-0.74, 1.02) |  |  |  |
| -0.09 (-0.67, 0.53) | **B** | 0.08 (-0.72, 0.92) | 0.11 (-0.64, 0.96) | -0.11 (-0.81, 0.62) | 0.05 (-0.81, 1.01) | 0.15 (-2.07, 2.10) | 0.72 (-2.13, 3.52) | 0.03 (-1.00, 1.16) |  |  |  |
| -0.16 (-0.73, 0.36) | -0.08 (-0.92, 0.72) | **C** | 0.04 (-0.39, 0.51) | -0.18 (-0.73, 0.32) | -0.02 (-0.52, 0.50) | 0.06 (-2.26, 2.19) | 0.65 (-2.29, 3.53) | -0.05 (-0.78, 0.78) |  |  |  |
| -0.19 (-0.74, 0.28) | -0.11 (-0.96, 0.64) | -0.04 (-0.51, 0.39) | **D** | -0.22 (-0.82, 0.30) | -0.06 (-0.56, 0.44) | 0.03 (-2.29, 2.14) | 0.61 (-2.31, 3.48) | -0.08 (-0.83, 0.70) |  |  |  |
| 0.02 (-0.35, 0.43) | 0.11 (-0.62, 0.81) | 0.18 (-0.32, 0.73) | 0.22 (-0.30, 0.82) | **E** | 0.16 (-0.48, 0.88) | 0.26 (-2.06, 2.35) | 0.83 (-2.08, 3.72) | 0.14 (-0.71, 1.10) |  |  |  |
| -0.14 (-0.84, 0.49) | -0.05 (-1.01, 0.81) | 0.02 (-0.50, 0.52) | 0.06 (-0.44, 0.56) | -0.16 (-0.88, 0.48) | **F** | 0.08 (-2.27, 2.22) | 0.65 (-2.27, 3.57) | -0.02 (-0.60, 0.60) |  |  |  |
| -0.24 (-2.25, 2.05) | -0.15 (-2.10, 2.07) | -0.06 (-2.19, 2.26) | -0.03 (-2.14, 2.29) | -0.26 (-2.35, 2.06) | -0.08 (-2.22, 2.27) | **G** | 0.61 (-1.40, 2.59) | -0.10 (-2.29, 2.32) |  |  |  |
| -0.81 (-3.68, 2.10) | -0.72 (-3.52, 2.13) | -0.65 (-3.53, 2.29) | -0.61 (-3.48, 2.31) | -0.83 (-3.72, 2.08) | -0.65 (-3.57, 2.27) | -0.61 (-2.59, 1.40) | **H** | -0.66 (-3.63, 2.31) |  |  |  |
| -0.12 (-1.02, 0.74) | -0.03 (-1.16, 1.00) | 0.05 (-0.78, 0.78) | 0.08 (-0.70, 0.83) | -0.14 (-1.10, 0.71) | 0.02 (-0.60, 0.60) | 0.10 (-2.32, 2.29) | 0.66 (-2.31, 3.63) | **K** |  |  |  |
| **LDL(mmol/L)** |  |  |  |  |  |  |  |  |  |  |  |
| **A** | 0.12 (-1.00, 1.26) | 0.36 (-0.71, 1.73) | 0.59 (-0.35, 1.69) | -0.03 (-1.11, 0.95) | 0.34 (-1.23, 1.98) | 0.34 (-1.44, 2.08) | -0.59 (-2.45, 1.15) |  |  |  |  |
| -0.12 (-1.26, 1.00) | **B** | 0.25 (-1.30, 2.05) | 0.46 (-0.95, 2.04) | -0.15 (-1.74, 1.34) | 0.22 (-1.70, 2.18) | 0.23 (-1.22, 1.61) | -0.71 (-2.22, 0.65) |  |  |  |  |
| -0.36 (-1.73, 0.71) | -0.25 (-2.05, 1.30) | **C** | 0.22 (-0.83, 1.17) | -0.41 (-1.77, 0.59) | -0.05 (-1.69, 1.49) | -0.06 (-2.34, 1.97) | -0.97 (-3.38, 1.00) |  |  |  |  |
| -0.59 (-1.69, 0.35) | -0.46 (-2.04, 0.95) | -0.22 (-1.17, 0.83) | **D** | -0.62 (-1.97, 0.45) | -0.27 (-1.56, 0.97) | -0.27 (-2.43, 1.67) | -1.19 (-3.42, 0.73) |  |  |  |  |
| 0.03 (-0.95, 1.11) | 0.15 (-1.34, 1.74) | 0.41 (-0.59, 1.77) | 0.62 (-0.45, 1.97) | **E** | 0.37 (-1.28, 2.14) | 0.38 (-1.64, 2.44) | -0.56 (-2.65, 1.47) |  |  |  |  |
| -0.34 (-1.98, 1.23) | -0.22 (-2.18, 1.70) | 0.05 (-1.49, 1.69) | 0.27 (-0.97, 1.56) | -0.37 (-2.14, 1.28) | **F** | 0.03 (-2.52, 2.39) | -0.89 (-3.42, 1.38) |  |  |  |  |
| -0.34 (-2.08, 1.44) | -0.23 (-1.61, 1.22) | 0.06 (-1.97, 2.34) | 0.27 (-1.67, 2.43) | -0.38 (-2.44, 1.64) | -0.03 (-2.39, 2.52) | **G** | -0.91 (-2.36, 0.45) |  |  |  |  |
| 0.59 (-1.15, 2.45) | 0.71 (-0.65, 2.22) | 0.97 (-1.00, 3.38) | 1.19 (-0.73, 3.42) | 0.56 (-1.47, 2.65) | 0.89 (-1.38, 3.42) | 0.91 (-0.45, 2.36) | **H** |  |  |  |  |
| **Triglycerides(mmol/L)** |  |  |  |  |  |  |  |  |  |  |  |
| **A** | 0.24 (-3.19, 3.70) | -5.39 (-10.82, 1.07) | -5.60 (-11.63, 1.28) | -0.14 (-2.37, 2.62) | -5.18 (-11.21, 1.69) | 1.66 (-3.37, 6.92) | -0.28 (-5.46, 5.01) | -5.14 (-11.83, 2.69) | -0.15 (-4.21, 4.50) |  |  |
| -0.24 (-3.70, 3.19) | **B** | -5.67 (-12.11, 1.46) | -5.85 (-12.78, 1.73) | -0.39 (-4.36, 4.07) | -5.42 (-12.28, 2.25) | 1.42 (-2.58, 5.31) | -0.52 (-4.52, 3.31) | -5.40 (-12.87, 2.96) | -0.39 (-5.66, 5.35) |  |  |
| 5.39 (-1.07, 10.82) | 5.67 (-1.46, 12.11) | **C** | -0.12 (-3.17, 2.51) | 5.30 (-0.63, 10.42) | 0.26 (-2.61, 3.10) | 6.97 (-1.18, 14.61) | 5.02 (-3.09, 12.70) | 0.26 (-4.04, 4.88) | 5.33 (-1.31, 11.49) |  |  |
| 5.60 (-1.28, 11.63) | 5.85 (-1.73, 12.78) | 0.12 (-2.51, 3.17) | **D** | 5.52 (-0.89, 11.32) | 0.42 (-2.50, 3.54) | 7.18 (-1.37, 14.94) | 5.16 (-3.29, 13.12) | 0.40 (-3.75, 5.26) | 5.52 (-1.60, 12.14) |  |  |
| 0.14 (-2.62, 2.37) | 0.39 (-4.07, 4.36) | -5.30 (-10.42, 0.63) | -5.52 (-11.32, 0.89) | **E** | -5.12 (-10.70, 1.44) | 1.76 (-4.19, 7.34) | -0.23 (-6.01, 5.50) | -5.08 (-11.50, 2.16) | 0.01 (-3.48, 3.53) |  |  |
| 5.18 (-1.69, 11.21) | 5.42 (-2.25, 12.28) | -0.26 (-3.10, 2.61) | -0.42 (-3.54, 2.50) | 5.12 (-1.44, 10.70) | **F** | 6.74 (-1.67, 14.65) | 4.73 (-3.87, 12.67) | 0.03 (-3.28, 3.56) | 5.10 (-2.00, 11.74) |  |  |
| -1.66 (-6.92, 3.37) | -1.42 (-5.31, 2.58) | -6.97 (-14.61, 1.18) | -7.18 (-14.94, 1.37) | -1.76 (-7.34, 4.19) | -6.74 (-14.65, 1.67) | **G** | -1.95 (-5.71, 2.07) | -6.70 (-15.43, 2.37) | -1.76 (-8.26, 5.25) |  |  |
| 0.28 (-5.01, 5.46) | 0.52 (-3.31, 4.52) | -5.02 (-12.70, 3.09) | -5.16 (-13.12, 3.29) | 0.23 (-5.50, 6.01) | -4.73 (-12.67, 3.87) | 1.95 (-2.07, 5.71) | **H** | -4.76 (-13.27, 4.55) | 0.21 (-6.43, 7.12) |  |  |
| 5.14 (-2.69, 11.83) | 5.40 (-2.96, 12.87) | -0.26 (-4.88, 4.04) | -0.40 (-5.26, 3.75) | 5.08 (-2.16, 11.50) | -0.03 (-3.56, 3.28) | 6.70 (-2.37, 15.43) | 4.76 (-4.55, 13.27) | **K** | 5.08 (-3.00, 12.22) |  |  |
| 0.15 (-4.50, 4.21) | 0.39 (-5.35, 5.66) | -5.33 (-11.49, 1.31) | -5.52 (-12.14, 1.60) | -0.01 (-3.53, 3.48) | -5.10 (-11.74, 2.00) | 1.76 (-5.25, 8.26) | -0.21 (-7.12, 6.43) | -5.08 (-12.22, 3.00) | **L** |  |  |
| **Notes**：95%CI=95% confidence intervals; FPG= fasting plasma glucose; HDL= High density lipoprotein; LDL= low density lipoprotein; A=Glibenclamide; B=Glimepiride; C= Pioglitazone; D=Rosiglitazone; E= Repaglinide; F= Metformin; G = Sitaglitin; H= Exenatide; I= Liraglutide; J= Acarbose; K= Benfluorex; L= Glipizide. | | | | | | | | | | | |
|  |  |  |  |  |  |  |  |  |  |  |  |
